# Supplementary material for: Brain size and neuron numbers drive differences in yawn duration across mammals and birds
Source: Commun Biol. 2021 May 6;4:503. doi: 10.1038/s42003-021-02019-y (PMC8102614; doi:10.1038/s42003-021-02019-y)
Supplement: Supplementary file 2 — Supplementary Information [file 42003_2021_2019_MOESM2_ESM.pdf]

Supplementary materials to:

Massen, Hartlieb, Martin, *et al.* (2021) Brain size and neuron numbers drive differences in yawn duration across mammals and birds. *Communications Biology*

Index:

Supplementary Note 1: Bayesian Power Analysis	**2**
Supplementary Note 2: Phylogenetic correlations independent of body size	**3**
Supplementary Note 3: Model comparison of yawn predictors	**4**
Supplementary Tables 1 & 2: Phylogenetic correlations tables	**5**
Supplementary Note 4: Neural density measures	**6**
Supplementary Figure 1	**7**

Supplementary Note 1: Bayesian Power Analysis

We used a Bayesian power analysis procedure to assess the probability of detecting a small effect (β = 0.10) using a Gamma GLMM, given the relatively small sample size ($N_{species}$ = 18) available for our mammal neuron count measures and the structure of the observed mammal phylogeny. We simulated 200 random datasets of yawn duration measures and body size adjusted neuron count z-scores for 18 species with 5 individual observations per species. The simulation assumed β_1 = 0.10 on the log Gamma scale for the effect of body size adjusted neuron count, with standard deviations σ = 0.3 and σ = 0.1 for the phylogenetic and residual species-level random effects respectively, and a global intercept $\beta_0 = 0.1$. The Gamma shape parameter was fixed $\alpha = 0.15$, with inverse scale parameter $\alpha * e^{-\mu_i}$ for the log-scale linear predictor μ_i of observation *i*. Power was assessed for the probability of detecting the true brain effect with $pp_+ \geq 0.95$ for a model estimated with weakly regularizing priors—$\beta \sim \mathrm{Normal}(0,1)$ and $\sigma \sim \mathrm{Exponential}(3)$. Power was estimated as the proportion of 200 random simulations for which $pp_+ \geq 0.95$ was satisfied. Please see the R code script for further details on this simulation procedure.

Supplementary Note 2: Phylogenetic correlations independent of body size

In the main text, we use the standard approaches of phylogenetic residuals and multiple regression to assess the effects of brain size and neuron count on yawn duration, adjusting for allometric scaling between the brain measures and body size. These results provide clear support for the effect of brain evolution on yawn duration, independent of body size evolution. However, to further examine the robustness of these findings, we also conducted additional analyses in which we first used Gaussian phylogenetic regressions to partial out the effects of body size on *both* brain and yawn duration prior to estimating their association. This approach relied on the simplifying assumption that yawn duration residuals are normally distributed, and we therefore used classical linear PGLS models for assessing the association among these residual values.

Consistent with the findings reported in the main text using more robust Bayesian methods, significant associations between yawn duration and brain measures were observed across mammals (brain size: $t = 2.90$, $p = 0.005$; neuron count: $t = 2.56$, $p = 0.02$; cortical neuron count: $t = 2.18$, $p = 0.04$) and birds (brain size: $t = 2.57$, $p = 0.01$; neuron count: $t = 2.42$, $p = 0.02$; cortical neuron count: $t = 2.50$, $p = 0.02$) after partialing body size from both the brain and yawn duration data. It should be noted that these simpler analyses provide stronger support for the effects of neuron counts on yawn duration independent of body size, as compared to those reported in the main text using more conservative Bayesian priors and appropriate non-Gaussian distributions to account for heteroscedasticity in yawn duration.

Supplementary Note 3: Model comparison of yawn predictors

As discussed in the main text, our brain size and neuron count measures were highly correlated in both the mammal and bird samples. This prevented us from clearly testing which of these predictors was more important for explaining variation in yawn duration across taxa, as the effect sizes of these measures were highly similar, particularly for mammals. Nonetheless, we also conducted an additional supplementary analysis to further probe whether any potential differences in the predictive value of these measures could be ascertained between models. Given that sample sizes varied across measures, these model comparisons were only conducted on species for which all measures were available. We used WAIC (see Methods section (Analyses) of main text) as a Bayesian information criterion for formal comparison, as explained above, with $\Delta\text{WAIC} \pm 2$ providing minimally sufficient evidence for a difference in predictive value between measures. Absolute rather than body size adjusted measures were used for the comparison because the adjusted models differed in whether phylogenetic residuals or multiple regression was used to account for body size. Comparing these models with and without body size included as an additional predictor would be inappropriate and confound the intended comparison.

Among mammals, model comparison provided no evidence for differences in the predictive value of brain size and total neuron count (ΔWAIC = -0.37 [1.11]), brain size and cortical neuron count (ΔWAIC = -0.26 [2.24]), nor of total neuron count compared to cortical neuron count (ΔWAIC = 0.12 [1.29]). Similarly, among birds, model comparison provided no support for differences between brain size and total neuron count (ΔWAIC = 0.50 [1.15]), brain size and pallium neuron count (ΔWAIC = 0.44 [1.50]), and total neuron count and pallium neuron count (ΔWAIC = -0.06 [0.58]).
It is important to emphasize that these findings should be interpreted with caution. These results are expected given the small sample sizes for neuron counts and high correlations among brain measures, which indicate that the size and count measures are providing largely redundant information across taxa. Future studies seeking to address this question should, therefore, consider using experimental methods to disentangle these measures, or otherwise selecting a lineage for which there is reason to suspect a priori that sufficient independent variation will occur in these measures to detect biologically meaningful differences.

Supplementary Tables 1 & 2: Phylogenetic correlations tables

Table S1. Mammals

	Body size	Brain size	Neuron count	Cortical neuron count
Body size	1			
Brain size	0.95	1		
Neuron count	0.90	0.98	1	
Cortical neuron count	0.81	0.93	0.96	1

Table S2. Birds

	Body size	Brain size	Neuron count	Cortical neuron count
Body size	1			
Brain size	0.96	1		
Neuron count	0.88	0.94	1	
Cortical neuron count	0.82	0.91	0.99	1

Supplementary Note 4: Neural density measures

Cortical and pallial neuronal densities were negatively associated with body size in both mammals (*r* = - 0.89, VIF = 4.13) and birds (*r* = - 0.85, VIF = 4.31). Overall brain neuronal density was also negatively correlated with body size, albeit with a much smaller effect size in mammals (*r* = -0.09, VIF = 1.07) as compared to birds (*r* = -0.90, VIF = 3.08). Consistent with this pattern, the relationship between brain neuronal density and pallial neuronal density was much stronger in birds (*r* = 0.98) than the relationship between brain neuronal density and cortical neuronal density in mammals (*r* = 0.13).

Among mammals, yawn duration was not clearly associated with brain neuronal density (β = -0.07 [0.11], 90% CI [-0.26, 0.12], p_{+}= 0.27, d = -0.24 [0.39]), nor with body size adjusted brain neuronal density (β = -0.05 [0.09], 90% CI [-0.20, 0.09], p_{+}= 0.39, d = -0.18 [0.31]). Yawn duration exhibited a negative association with cortical neuronal density (β = -0.28 [0.10], 90% CI [-0.45, -0.10], p_{+}= 0.01, d = -1.01 [0.36]), but this association disappeared once body size was adjusted for (β = -0.03 [0.13], 90% CI [-0.25, 0.19], p_{+}= 0.67, d = -0.11 [0.46]).

Yawn duration also had a moderately sized association with brain neural density across birds (β = -0.15 [0.10], 90% CI [-0.31, 0.02], p_{+}= 0.07, d = -0.49 [0.33]), but no association was found after adjusting for body size (β = 0.01 [0.09], 90% CI [-0.14, 0.15], p_{+}= 0.52, d = 0.02 [0.30]). Pallial neuronal density also did not clearly associate with yawn duration (β = -0.09 [0.11], 90% CI [-0.28, 0.08], p_{+}= 0.20, d = -0.31 [0.36]), nor when adjusting for body size (β = 0.05 [0.09], 90% CI [-0.11, 0.21], p_{+}= 0.70, d = 0.17 [0.32]). Taken together, these results suggest that there is no unique relationship between yawn duration and either brain or cortical/pallial neuronal density, with the observed associations for unadjusted measures being entirely accounted for by variation in body size.

Supplementary Figure 1

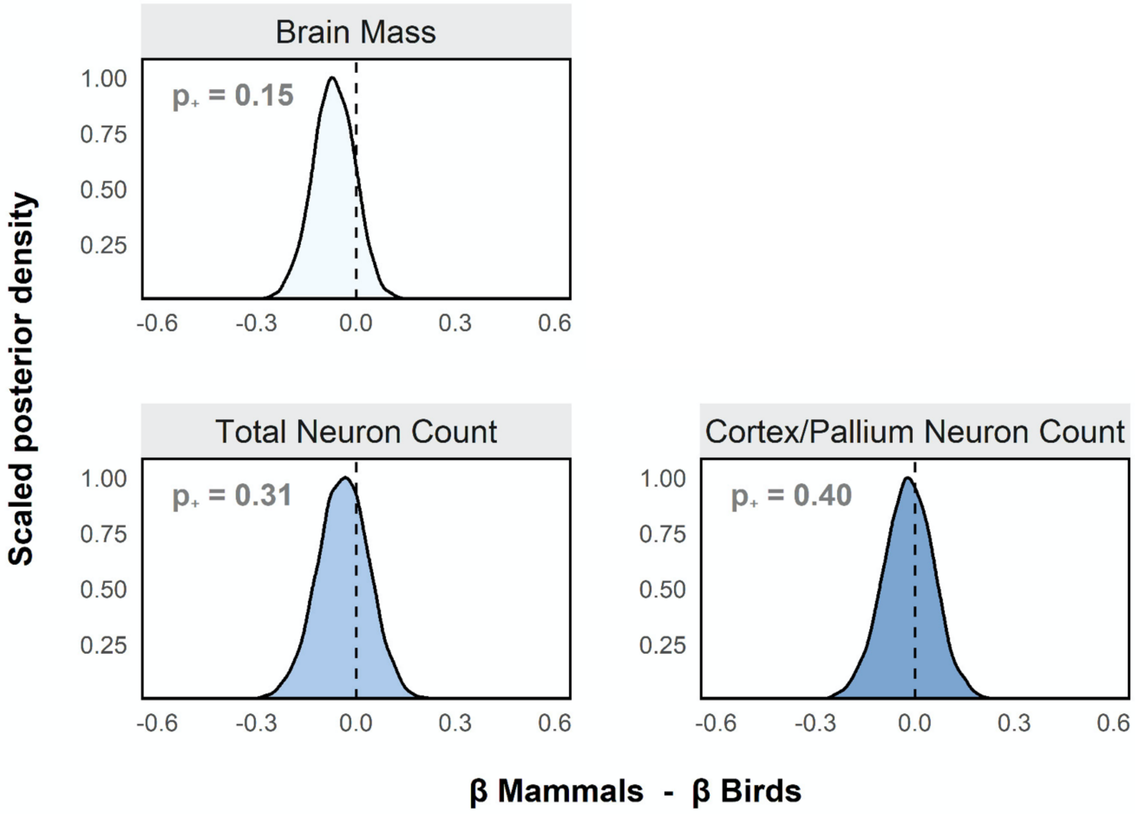

Fig. S1. *Posterior differences of brain size effects on yawn duration between mammals and birds*. Posterior distributions are shown for the difference of mammal and avian yawn duration regression coefficients (β) across each brain size measure. The dotted vertical line at 0 demarcates support for a larger association between yawn duration and brain size in mammals (+0) or birds (-0). The posterior probability supporting a larger effect on yawn duration for mammals compared to birds (p_+) is shown in each plot. These estimates are marginalized over phylogenetic autocorrelation and thus provide unbiased comparisons between our samples, while also accounting for all statistical uncertainty in the coefficients for each clade.
