# Supplementary material for: Brain size and neuron numbers drive differences in yawn duration across mammals and birds
Source: Commun Biol. 2021 May 6;4:503. doi: 10.1038/s42003-021-02019-y (PMC8102614; doi:10.1038/s42003-021-02019-y)
Supplement: Supplementary file 3 — Reporting Summary [file 42003_2021_2019_MOESM3_ESM.pdf]

nature research

Corresponding author(s): jorgmassen@gmail.com

Last updated by author(s): Mar 10, 2021

Reporting Summary

Nature Research wishes to improve the reproducibility of the work that we publish. This form provides structure for consistency and transparency in reporting. For further information on Nature Research policies, see our Editorial Policies and the Editorial Policy Checklist.

Statistics

For all statistical analyses, confirm that the following items are present in the figure legend, table legend, main text, or Methods section.

n/a	Confirmed	
☐	☒	The exact sample size (*n*) for each experimental group/condition, given as a discrete number and unit of measurement
☐	☒	A statement on whether measurements were taken from distinct samples or whether the same sample was measured repeatedly
☐	☒	The statistical test(s) used AND whether they are one- or two-sided<br>*Only common tests should be described solely by name; describe more complex techniques in the Methods section.*
☐	☒	A description of all covariates tested
☐	☒	A description of any assumptions or corrections, such as tests of normality and adjustment for multiple comparisons
☐	☒	A full description of the statistical parameters including central tendency (e.g. means) or other basic estimates (e.g. regression coefficient) AND variation (e.g. standard deviation) or associated estimates of uncertainty (e.g. confidence intervals)
☒	☐	For null hypothesis testing, the test statistic (e.g. *F*, *t*, *r*) with confidence intervals, effect sizes, degrees of freedom and *P* value noted<br>*Give P values as exact values whenever suitable.*
☐	☒	For Bayesian analysis, information on the choice of priors and Markov chain Monte Carlo settings
☐	☒	For hierarchical and complex designs, identification of the appropriate level for tests and full reporting of outcomes
☐	☒	Estimates of effect sizes (e.g. Cohen's *d*, Pearson's *r*), indicating how they were calculated

Our web collection on statistics for biologists contains articles on many of the points above.

Software and code

Policy information about availability of computer code

Data collection	n.a.
Data analysis	The 'brms' package for R statistical environment was employed for all analyses, which interfaces with the Stan statistical programming language

For manuscripts utilizing custom algorithms or software that are central to the research but not yet described in published literature, software must be made available to editors and reviewers. We strongly encourage code deposition in a community repository (e.g. GitHub). See the Nature Research guidelines for submitting code & software for further information.

Data

Policy information about availability of data

All manuscripts must include a data availability statement. This statement should provide the following information, where applicable:

- Accession codes, unique identifiers, or web links for publicly available datasets
- A list of figures that have associated raw data
- A description of any restrictions on data availability

All data are made available on an online repository; i.e. DataverseNL. This "Dataverse" contains:
1. Source data for brain measures with additional references, as well as source data for each yawn, separately for mammals and birds. (Link for reviewers: https://dataverse.nl/privateurl.xhtml?token=1aa48c2a-5b16-46e9-ba22-985d47a69d0d)
2. Database of all yawn videos collected by the authors, or with permission from original recorder(s) (birds; link for reviewers: https://dataverse.nl/privateurl.xhtml?token=b20c12a2-7bc7-449b-9882-ce89a3581307; mammals; link for reviewers: https://dataverse.nl/privateurl.xhtml?token=c09b19d2-6361-4bae-b336-ae88887268ca).
3. Phylogenetic trees for both mammals and birds, specific to the samples of our analyses. (link for reviewers: https://dataverse.nl/privateurl.xhtml?

token=88a46634-5098-4be2-b0f8-8652343b3d85)
4. Final data files (csv). (link for reviewers: https://dataverse.nl/privateurl.xhtml?token=d4e990bb-d5d4-487e-ad47-11e260b76356)
5. R scripts. (link for reviewers: https://dataverse.nl/privateurl.xhtml?token=8f7458b5-a999-4cc6-a673-f4ced801c8f5)

Field-specific reporting

Please select the one below that is the best fit for your research. If you are not sure, read the appropriate sections before making your selection.

☐ Life sciences ☐ Behavioural & social sciences ☒ Ecological, evolutionary & environmental sciences

For a reference copy of the document with all sections, see nature.com/documents/nr-reporting-summary-flat.pdf

Ecological, evolutionary & environmental sciences study design

All studies must disclose on these points even when the disclosure is negative.

Study description	We collected and analyzed the duration of 1,291 yawns from 697 individuals across 101 different species (55 mammals; 46 birds). Per species we had at least 2 yawns from at least 2 individuals. Bayesian multilevel phylogenetic models were used to investigate the associations between yawn duration and several brain measures of these species while accounting for species' phylogenetic history
Research sample	The original sample of yawns consisted of 1557 yawns (831 mammal yawns and 726 bird yawns), of 810 individuals (523 mammals, 287 birds) of 110 species (60 mammal species and 50 bird species), but was reduced due to several exclusion criteria (see below). The vast majority of brain data was collated from published reports (data sources are provided). Neuron numbers for ten species (see below) were assessed in this study. These birds were selected to represent distantly related avian groups and size categories and were purchased from local breeders or from local hunters. The Common kestrels were injured animals obtained from a local wildlife rescue center.
Sampling strategy	With regard to the yawn videos, we aimed to sample those species of which most brain measures were known. Apart from that sampling was opportunistic; i.e. species that were available at local zoos, research labs, or rescue centres, or of which we could find videos while yawning online. As to the additional neuroanatomical examination, two to four individuals per species were used. This is a sufficient number, because intraspecific variation in brain mass and neuron numbers is much smaller than interspecific variation in these traits. The Red-breasted goose (Branta ruficollis) was an exception, because only one individual was available.
Data collection	Videos of yawning birds and mammals were collected from online sources (YouTube, shutterstock, gettyimages, footage framepool, vine, 123rf, istockphoto), from videos provided by colleagues or zoos, or were collected by us using handheld cameras filming the different animals in zoos and research institutes by JJMM, MH, EL, JH, and ACG
Timing and spatial scale	Data collection took place between March 2017 and December 2019, in an opportunistic, ad libitum manner; i.e. whenever we found specific species to be present at a specific location we went there and started filming the subjects, waiting for them to yawn. In addition, videos of yawning birds and mammals were collected from online sources (YouTube, shutterstock, gettyimages, footage framepool, vine, 123rf, istockphoto), throughout the sampling period.
Data exclusions	We excluded: a) all videos for which the yawn could not be validated, b) all species of which we did not have at least 2 different yawns; i.e. also from two different individuals, c) two additional species, domestic dogs and chicken, because brain measures of these species tend to vary substantially per breed, and d) an additional 5 domesticated species, because due to their domestication they have suffered different evolutionary constraints, the length of their phylogenetic distances are unclear and it is therefore difficult to correctly place them in a phylogenetic tree, which was necessary for our phylogenetic models.
Reproducibility	All yawning videos were then checked for validity (is it a yawn yes/no) by, and all doubtful cases were subsequently checked. In addition, a random selection of 16.3% of all yawn videos were recoded by a second researcher and inter-rater reliability was excellent (Spearman's rho combined = 0.9718691, p < 2.2e-16)
Randomization	Data collection was done through haphazard sampling
Blinding	While coding the length of yawns the speficic researcher was 'blind' to the actual brain mass and neuron numbers of the specific species, although she could obviously make an educated guess with regard to comparisons between species. Therefore, we specifically tested inter-rater reliability and this was near perfect (see above).

Did the study involve field work? ☐ Yes ☒ No

Reporting for specific materials, systems and methods

We require information from authors about some types of materials, experimental systems and methods used in many studies. Here, indicate whether each material, system or method listed is relevant to your study. If you are not sure if a list item applies to your research, read the appropriate section before selecting a response.

Materials & experimental systems

n/a	Involved in the study
☐	☒ Antibodies
☒	☐ Eukaryotic cell lines
☒	☐ Palaeontology and archaeology
☐	☒ Animals and other organisms
☒	☐ Human research participants
☒	☐ Clinical data
☒	☐ Dual use research of concern

Methods

n/a	Involved in the study
☒	☐ ChIP-seq
☒	☐ Flow cytometry
☒	☐ MRI-based neuroimaging

Antibodies

Antibodies used	anti-NeuN mouse monoclonal antibody (clone A60, Sigma-Aldrich, MAB377)
Validation	The antibody was characterized by Western blotting with chick brain – Mezey, S. et al. Postnatal changes in the distribution and density of neuronal nuclei and doublecortin antigens in domestic chicks (Gallus domesticus). J. Comp. Neurol. 520, 100-116 (2012).<br>and shown by immunocytochemistry that it reliaby stains neurons in chicken – Ngwenya A, Nahirney J, Brinkman B, Williams L, Iwaniuk AN: Comparison of estimates of neuronal number obtained using the isotropic fractionator method and unbiased stereology in day old chicks (Gallus domesticus). J Neurosci Methods 2017, 287:39-46.<br>and many other birds – Olkowicz, S. et al. Birds have primate-like numbers of neurons in the forebrain. Proc. Natl. Acad. Sci. U. S. A. 113, 7255-7260 (2016).

Animals and other organisms

Policy information about studies involving animals; ARRIVE guidelines recommended for reporting animal research

Laboratory animals	None
Wild animals	Common kestrel, Falco tinnunculus; Diamond dove, Geopelia cuneata; Red-breasted goose, Branta ruficollis; Mallard, Anas platyrhynchos; Greylag Goose, Anser anser, Helmeted Guineafowl, Numida meleagris; Indian peafowl, Pavo cristatus; Common ostrich, Struthio camelus; Greater rhea, Rhea americana; Emu, Dromaius novaehollandiae; Elegant crested Tinamou, Eudromia elegans
Field-collected samples	Mallard, Anas platyrhynchos; Greylag Goose, Anser anser; Common kestrel, Falco tinnunculus
Ethics oversight	All procedures were approved by Institutional Animal Care and Use Committee at Charles University in Prague, Ministry of Culture (Permission No. 47987/2013) and Ministry of the Environment of the Czech Republic (Permission No. 53404/ENV/13-2299/630/13).

Note that full information on the approval of the study protocol must also be provided in the manuscript.
